# COVID-19 and the Political Economy of Mass Hysteria

**DOI:** 10.3390/ijerph18041376

**Published:** 2021-02-03

**Authors:** Philipp Bagus, José Antonio Peña-Ramos, Antonio Sánchez-Bayón

**Affiliations:** 1 Department of Applied Economics I, History and Economic Institutions and Moral Philosophy, Social and Legal Sciences Faculty, Rey Juan Carlos University, 28033 Madrid, Spain; 2 Faculty of Social Sciences and Humanities, Universidad Autónoma de Chile, Providencia 7500912, Chile; japramos@ugr.es; 3 Department of Business Economics (ADO), Applied Economics II and Fundamentals of Economic Analysis, Social and Legal Sciences Faculty, Rey Juan Carlos University, 28033 Madrid, Spain

**Keywords:** mass hysteria, nocebo effects, contagion, mass media, social media, public health, law and economics, political economy, groupthink, culture of fear, emotional contagion, anxiety, policy error, COVID-19

## Abstract

In this article, we aim to develop a political economy of mass hysteria. Using the background of COVID-19, we study past mass hysteria. Negative information which is spread through mass media repetitively can affect public health negatively in the form of nocebo effects and mass hysteria. We argue that mass and digital media in connection with the state may have had adverse consequences during the COVID-19 crisis. The resulting collective hysteria may have contributed to policy errors by governments not in line with health recommendations. While mass hysteria can occur in societies with a minimal state, we show that there exist certain self-corrective mechanisms and limits to the harm inflicted, such as sacrosanct private property rights. However, mass hysteria can be exacerbated and self-reinforcing when the negative information comes from an authoritative source, when the media are politicized, and social networks make the negative information omnipresent. We conclude that the negative long-term effects of mass hysteria are exacerbated by the size of the state.

## 1. Introduction

Public healthcare systems form a vital part of the welfare state. Indeed, it is generally taken for granted that one main purpose of the modern welfare state is to improve public health. It is supposed that the state positively contributes to public health. In this article, we question this narrative in relation to the phenomenon of mass hysteria. We analyze how the modern state influences the development and extension of mass hysteria, arguing that the state exacerbates this phenomenon with adverse consequences for public health. By developing a political economy of mass hysteria, we fill an apparent gap in the literature. There have been many illuminating studies on psychological issues related to the phenomena of mass hysteria. As a consequence of the COVID-19 crisis, there have been several studies examining the adverse psychological effects of state-imposed lockdowns [1,2,3,4]. There are also studies that examine the contribution of digital media and the internet to anxiety [5,6], emotional contagion [7,8], anxiety transmissions [9,10], and nocebo effects [11,12]. However, to our knowledge, there has been no study that analyzes how different political institutions and the state affect the development and extension of mass hysteria. The interplay of media, science, politics, and public is a real research gap [13]. Building on the psychology related to the phenomenon of mass hysteria, we develop a political economy of mass hysteria deriving important insights from a public health perspective.

In a multidisciplinary analysis (beyond Law and Economics or Sociological Economics), we show that the size of the state exacerbates the negative consequences of mass hysteria. As a conceptual framework, we use a public choice approach to political institutions and comparative political economy based on economic principals. Developing a political economy of mass hysteria is important because it is important to examine how the political system influences the likelihood and development of mass hysteria. This is because mass hysteria can lead to policy mistakes that have tragic public health consequences. While there are important limits on the potential growth of a mass hysteria in a limited minimal state, the welfare state of the 21st century combined with a sensationalist mass media is likely to increase the havoc created by mass hysteria. In this context, we comment on the illustrative case of the COVID-19 crisis.

In the second section, we present a short history of mass hysteria. In this context, we also review the literature, theoretical and empirical, on mass psychogenic illness. In the following section, we present the importance of nocebo effects, explain how a mass hysteria evolves, and analyze how negative information and anxiety contagion can contribute to mass hysteria in the information age. In the discussion section, we analyze the factors that limit and reduce mass hysteria in a free market setting. Moreover, in the same section, we show that these limiting mechanisms not only are disturbed by state action, but also examine the reasons why the state is likely to foster mass hysteria. We conclude that collective hysteria may have contributed to policy errors during the COVID-19 pandemic that were detrimental to public health. In order to prevent the repetition of such policy errors, one should be aware of the political economy of mass hysteria developed in this article.

## 2. Literature, History and Methods

In this article, we rely on case studies on mass hysteria, psychological research, and theoretical comparative political economy. Our article focuses on empirical data on mass hysteria and research related to public health and anxiety contagion. We analyze the role of nocebo effects in mass hysteria and research on the negativity bias of the human mind. On this basis, we develop a comparative political economy of mass hysteria. We compare the conditions for a mass hysteria to develop in a modern welfare state with the conditions in a limited, minimal state. Note that these differences between the welfare state and a minimal state apply a fortiori to a comparison between the modern state and a private law society, because in a private law society, the state is non-existent [14,15,16].

In a mass hysteria, people of a group start to believe that they might be exposed to something dangerous, such as a virus or a poison. They believe a threat to be real because someone says so, or because it fits their experience. Due to the threatening delusion, a large group of people gets collectively very upset. In other words, a threat, whether real or imaginary [17], causes collective anxiety [18]. The group members may even start to feel sick. Group members might also get symptoms of sickness including weakness, headaches, or a choking feeling, which are propagated to other persons. When a mass hysteria causes physical symptoms, it is called mass psychogenic illness or epidemic hysteria. The symptoms are caused by the stress and anxiety people experience due to the perceived threat [19]. Mass hysteria is infectious [20] and may be a contributing and amplifying factor in real epidemics.

While there is—to our knowledge—no literature on the political economy of mass hysteria, the literature on mass psychogenic illness is rich and focuses on empirical analyses of specific cases. Kerckhoff [21] analyzed the case of sickness that spread among workers of a plant due to the belief in a poisonous insect. McGrath [22], reviewing cases of mass hysteria, found that persons of low status in high stress situations after a triggering dramatic event are most responsive to mass psychogenic illness. Schmitt and Fitzgerald [23] analyzed eight cases of mass psychogenic illness among workers. They found that low income, dissatisfaction with superiors, lack of support, and unclear work assignments led to a higher average number of reported symptoms. Singer [24] points out that victims of mass psychogenic illness are really sick even though there is no toxin. Singer believes that mass psychogenic illness occurs more often than we recognize as it may appear simultaneously with physical progenitors of illness and we only count “pure” cases of mass psychogenic illness.

There also exists more theoretical orientated literature related to mass psychogenic illness. Pennebaker [25] argued that in order to reduce the possibility of mass psychogenic illness, the true causes of anxiety must be diminished. Singer et al. [26] discussed the role of social comparison as a cause of mass psychogenic illness emphasizing the role of stress. Freedman [27] discussed theories of contagion in reference to mass psychogenic illness claiming that contagion, conformity, and emergent norms may play a role in spreading the hysteria. Stahl [28] used labeling theory, emergent norms, and coping theories to explain and understand mass psychogenic illness. Kerckhoff [29] emphasized the importance of collective tension in the origination of mass psychogenic illness.

As can be seen in the literature review, the literature reviewed deals with outbreaks of mass psychogenic illness mostly in localized settings of schools or companies. Unfortunately, there are no studies on the possibility of more widespread or even global cases of mass hysteria. However, the digital age of a global mass and social media raises the possibility of such a phenomenon. Our study of the political economy of mass hysteria draws on the well-established psychological phenomenon of mass hysteria but applies it to a new and innovative context for which no literature yet exists. More specifically, it analyzes how the political system can influence the likelihood and spread of mass hysteria in a digitized and globalized world.

The empirical evidence of mass hysteria, i.e., collective anxiety due to a perceived threat, dates back at least to the Middle Ages [30,31] and continues to numerous cases in modern times [32,33,34,35]. One of the most famous cases is a hysteria that developed after a radio play written by Orson Welles, *War of the Worlds*, was broadcasted in 1938. In the radio play, an attack from Martians on the Earth occurs. Some of the listeners, possibly still under the suspense of the recent Munich agreement the same year, allegedly fell to panic, thinking they were really under attack by Martians [36].

Another interesting, more recent case are the effects of an episode of the Portuguese TV show *Strawberries with Sugar* [37]. In the show, the characters got infected with a life-threatening virus. After the episode had been broadcasted, more than three hundred Portuguese students fell ill. They reported symptoms similar to the ones that the TV show characters had experienced. Among these symptoms were rashes and difficulties to breathe. As a result of these symptoms, several schools in Portugal actually closed. However, an investigation of the Portuguese National Institute for Medical Emergency concluded that the virus did not exist in reality and that the symptoms were caused by the anxiety watching the show, i.e., the symptoms were caused by mass hysteria.

There is another recent case of mass hysteria connected to a virus. On the Emirates flight 203 in September 2018, some passengers were showing flu-like symptoms [38]. When other passengers observed these symptoms, they started to feel sick as well, and a panic broke out. The panic reached such an extent that the whole flight was quarantined once it had reached New York. The investigation after the incident showed that only a few passengers actually had seasonal flu or a common cold. Indeed, diseases are an ideal ground for mass hysteria to develop.

## 3. Nocebo Effects and the Evolution of Mass Hysteria

### 3.1. Nocebo Effects

It is well known that in addition to placebo effects, so-called “nocebo” effects also exist [39]. Due to the placebo effect, a person recovers from an illness because they expect to recover. When a person suffers from a nocebo effect, on the contrary, they get ill just because they expect to become ill. An intriguing and famous case of a nocebo effect is the case of a man who tried to commit suicide [40]. The man was involved in a clinical study taking an experimental drug. In order to kill himself, he swallowed twenty-nine capsules of the drug, believing he would not survive. However, the capsules that he was taking were placebos, as he was a member of the control group in the clinical study. Believing that he was going to die, he developed serious symptoms and arrived at the hospital with extremely low blood pressure. When, finally, the doctor directing the medical trial arrived, the doctor told the patient that he had swallowed placebos. As a consequence, the man recovered within fifteen minutes.

Due to the nocebo effect, the expectation to become ill can cause real symptoms in a self-fulfilling prophecy. In this way, a mass hysteria may develop when people believe they will become ill. Anxiety and fear contribute to this process [41]. Indeed, during the Spanish flu in the wake of World War I, panic contributed to a mass hysteria and deaths that otherwise would not have occurred, because panic can have adverse health effects on ill persons [42]. Once some people develop a hysteria, it can easily spread to other people because fear and anxiety are contagious [43].

In principle, pseudo-infectious people could be “cured” by mere information. In this way, a mass hysteria could be prevented from becoming a burden on the health system. As discussed below in detail, the problem in a mass hysteria is that there are reasons both media and the state may actively contribute to the contagion of fear and spread biased information. In other words, the doctor telling the patient that they had swallowed placebos never arrives.

### 3.2. Mass Hysteria, Irrationality, Biases, and COVID-19

Hysteria can not only cause people to suffer symptoms [44]. Hysteria, be it collective or not, can make people behave in ways that other persons unaffected by the hysteria would likely consider to be irrational. Under the illusion of a non-existing or highly exaggerated threat, people act in ways that in the absence of the illusion would seem absurd. Alternatively, behavior in mass hysteria can be considered biased. Psychological research on risk perception has found that some mental rules that people apply in an uncertain world create persistent and important biases. Biased media coverage, incomplete and asymmetric information, personal experiences, fears, inability to understand and interpret statistics, and other cognitive biases lead to distorted risk judgments. Risk perceptions may be particularly biased when risks are viewed as unfair, uncontrollable, unknown, frightening, potentially catastrophic, and impacting future generations [45,46]. Whether we call people’s behavior in a mass hysteria “irrational” or “biased” is not essential to the purpose of this article, as we purport to develop a political economy of mass hysteria. We examine the extent to which the state influences the development of mass hysteria and the “irrational” or “biased” behavior typical of it.

If, and to what extent, the world has been suffering from mass hysteria or mass psychogenic illness during the COVID-19 crisis is open to future research, even though some observers have made that claim [47]. It is clear, in any case, that the population has been under tremendous psychological strain during the COVID-19 crisis. Especially lockdowns have contributed to a surge in anxiety and stress, which are important ingredients for the development of mass hysteria [48]. In a survey conducted in the US from 24 to 30 June, 40.9% of participants reported at least one adverse mental health condition, and 10.7% reported to have considered suicide seriously in the last 30 days [49]. Additionally, the frequency of alcohol consumption during lockdowns increased 14% in the US [50]. At least some anecdotical evidence points to the possibility of mass hysteria as manifested by the hoarding of toilet paper and other essentials, the masked driving of single persons in their cars, and people virtually not leaving their houses, not even for a walk, even though the risk of being infected outdoors with physical distancing is minuscule. Similarly, some people have been scared by SARS-CoV-2 to an extent not easily explainable by their own miniscule risk of death from it [51]. It seems that many people believed in the existence of a killer virus far more mortal than SARS-CoV-2 actually is, as can be seen in Table 1.

Another indicator of overestimation of the threat is the number of deaths. As of 22 January 2021, 2.1 million deaths have been classified as COVID-19-related [53]. However, other diseases are equally or even far more deadly and do not trigger panic or unprecedented government intervention. In other words, the probability to die from COVID-19 is not only very low in absolute terms, but it is also lower than the probability to die from other diseases. It is true that the majority of these other diseases are not as infectious as SARS-CoV-2. This fact has contributed to the panic and led to government interventions that do not occur with other diseases that are even more deadly than COVID-19. The ten leading causes of death worldwide can be seen in Table 2.

Investigating the possibility and extension of a mass hysteria related to COVID-19 is beyond the scope of this article. In this article, we analyze a more fundamental question, namely, the role of the modern welfare state in mass hysteria. There can certainly be mass hysteria without the state in a private law society or within the context of a minimal state. This possibility exists due to the negativity bias of the human brain [55], which makes people vulnerable to delusions. Due to biological evolution, we focus on bad news as it may represent a possible threat [56]. Focusing on negative news and feeling a loss of control [57] may cause psychological stress that can develop into a hysteria and propagate to a larger group.

In a society with a minimal state, negative news may start such hysteria. Due to the negative news, some people start to believe in a threat. This threat evokes fear and begins to spread in society. Symptoms can also spread. Le Bon [58] called the spread of emotions through groups “contagion”. Once anxiety has spread and the majority of a group behaves in a certain way, there is the phenomenon of conformity, i.e., social pressure makes individuals behave in the same way as other members of the group. In the end, there may be a phenomenon that has been called emergent norms [59]: when a group establishes a norm, everyone ends up following that norm. For example, if a group decides to wear masks, everyone agrees to that norm. Emergent norms may explain the later stages of contagion. Contagion by fear can lead people to overreact strongly in a situation, even in a minimal state. Nonetheless, in a minimal state, there exist certain self-corrective mechanisms and limits that make it less likely for a mass hysteria to run out of control.

## 4. Discussion of Amplifiers and Attenuators of Mass Hysteria: Minimal State vs. Welfare State

There are several corrective mechanisms and limits for a mass hysteria. There exist well-known strategies to reduce anxiety, stress, and fear which individuals can employ in a society with a minimal state. Releasing tension from one´s body through sports and exercises helps to limit psychological stress [60]. Furthermore, it is essential to find distractions from the negative news and to socialize. Without state restrictions, such distractions abound.

Hysteria may cause people to inflict harm on themselves and innocent bystanders. In a society with a minimal state, there exists an essential limit to the destruction resulting from mass hysteria, which is the enforcement of private property rights, which in theory is the only task of a minimal state [61,62]. Most importantly, in such an institutional environment, there does not exist an institution that is powerful enough to massively violate private property rights, perhaps with the possible exception of the minimal state converting itself into a welfare state.

In addition, while anyone in a hysteria related to public health may voluntarily close their own business, wear a mask, or stay at home, in a minimal state, no one can use coercion to force others who are healthy and do not succumb to the hysteria to close their businesses, wear masks, or quarantine. A minority can just ignore the collective panic and continue to live their normal lives, because they are free to do so. Such a minority can be an example and a wake-up call to those that do succumb to the collective hysteria or are close to doing so. This minority may be especially attractive to borderline cases. Suppose that a small group of people during a collective health hysteria continues to go shopping, to work, to socialize, and breathe freely and does not fall ill (massively and fatally). Having this example, the anxiety of observers may fall. Observers may follow the example, and the group of hysterics shrinks. It is one of the core characteristics of decentralized systems that they allow for competition, error detection, and correction [63,64,65]. If the people that ultimately become role models for others through their interaction become ill and die, the panic would be confirmed. However, if there is really a hysteria and the threat is imagined or exaggerated, the fortune of the role models will be on average much better than is expected by those that succumbed to the hysteria. A sufficient number and variety of role models allows observers to correct and adjust their expectations [66].

Thus, there exist important limits for a mass hysteria to harm life and liberty in a minimal state. Moreover, natural mechanisms that reduce stress, anxiety, and hysteria can operate freely. Decentralized competition for solutions alleviates pressure to conform and facilitate breaking out of hysteria. Competition allows discovering information about the real danger of the perceived threat [67]. While the havoc inflicted by collective hysteria is limited by the protection of private property rights in a private law society or a minimal state, such limits can be easily overcome by a modern welfare state. In fact, a well-organized group [68] that has been infected by collective hysteria may be in charge of the state or get to control the state apparatus. In such a position, this group can impose measures on the rest of the population, inflicting almost unrestricted harm. It must be taken into account here that a welfare state can also be a state which is bound by the rule of law [69,70] and in which repressive interventions by the executive can be lifted by the judiciary. However, there exists the danger that in a collective panic, the protection of basic liberties guaranteed in constitutions will be abrogated by emergency measures and the judiciary will succumb to the mass hysteria, failing to lift the repressive interventions. The empirical evidence during the COVID-19 crisis demonstrates that basic liberties were not defended in welfare states [71]. In general, the greater the coercive power of the state, the more harm can be inflicted on society in a mass hysteria. It could be argued that infection with a virus would constitute a negative technological external effect [72]. However, the only task of a minimal state is to protect private property rights. It is not the task of the minimal state to protect its citizens against all risks of life, such as getting a cold or the seasonal flu [73]. In a minimal state, citizens are free to decide which risks they want to assume, be they driving a car, doing bungee jumping or engaging in social interaction. Indeed, the state´s attempt to reduce infection rates in the form of mandatory face masks, the shutdown of businesses or shelter in place orders does violate the private property rights that the minimal state is supposed to defend and may produce negative externalities in form of depressions, alcoholism or suicides.

While in a private law society and in a minimal state, there are mechanisms that help to limit and reduce mass panics, collective hysteria may be exacerbated by a powerful welfare state for several reasons.

First, the state has the power to diminish and prohibit those activities that do reduce fear and anxiety, such as sports, diversion, and socializing. During the COVID-19 crisis, states used their coercive power to impose social isolation, thereby contributing to anxiety [74] and psychological strain, both ingredients that spur mass hysteria. In order to shield against biopsychological infections, the population should exercise regularly, have quality sleep, exercise regularly, have a balanced nutrition, and maintain a strong connection with other people. Governments around the world mandated lockdowns and masks during the COVID-19 crisis, making it more difficult for citizens to do any of these things. More specifically, social distancing imposed by governments reduces strong social connections, and mandatory masks prevent expressing friendliness and compassion, thereby decreasing psychological resilience [75].

Second, the state, by its very nature, takes a centralized approach to solving problems. It is true that a welfare state is not necessarily a completely centralized state. The USA and the Federal Republic of Germany—both of which are welfare states—have substantial federal structures, and these federal structures can lead to competition in regulations and in dealing with the source of a mass hysteria, which leads to better solutions. Moreover, the existence of competing states on the international level allows experimenting with different solutions. During the COVID-19 crisis, for instance, the approach of Sweden provided evidence of the results of alternatives approaches [76]. Generally speaking, the more decentralized the political structure, the more intense the possible competition.

In any case, the state, by its very nature, deals with the source of a hysteria, such as the perceived threat of a deadly virus, in a centralized way. The state is the monopolist of coercion in a given territory [77]. As the state imposes its solution to the problem, there is no or only very limited experimentation of alternative ways to solve the problem. People that oppose the state´s approach to the problem because they have not succumbed to the hysteria are suppressed. They cannot demonstrate alternative ways to solve the “crisis”, as these alternative ways are prohibited by the state. When alternatives are ruled out, groupthink increases. Groupthink is a psychological force that fosters consensus, suppressing dissent and the evaluation of alternatives to the collective narrative. Groupthink has been considered responsible for political fiascos such as the Vietnam War or the Watergate coverup by Irving Janis [78]. Lockdowns during the COVID-19 crisis may be another fiasco candidate as their effectiveness is disputed [79,80]. Group pressure can modify and distort judgments as has been shown by the Ash experiments [81]. The human inclination toward conformity aids hysteria spread. Indeed, groupthink helps to explain the phenomenon of mass hysteria [82]. Mass hysteria can be considered to be a form of groupthink [83]. Due to group pressure and groupthink, hysteria feeds itself, as no alternatives are shown to people. The information necessary to address the problem cannot be generated in a decentralized way in the market, which is a problem inherent in socialism [84].

Third, in a modern welfare state, the media may be politicized. This politization restricts the existing competition between the media. There are several mechanisms that channel and even restrict media competition. News outlets and social media platforms may develop close relationships with the state. The state regulates media, and it may also own media outlets directly, such as public TV or radio channels. The state typically also requires licenses for certain media to operate. In general, new outlets and platforms may look for the goodwill of state agencies. Moreover, government officials are often used as a source of news, leading to a statist bias. While objectivity would require presenting both sides of a story, in times of crisis, politicians often present both sides of the story. Another form of indirect state influence in the media is that they are staffed with people who were educated in state or state-licensed schools, reinforcing the statist bias in media. News agencies and social media platforms connected with the state may engage in and promote massive negative news campaigns. Negative news sells. The media have the incentive to portray danger. The story of the government as a hero who provides a resolution to threats is very marketable [85].

In fact, mass media spread panic by presenting SARS-CoV-2 as an unprecedented threat [86]. Information seeking on the internet was associated with more symptoms during the COVID-19 crisis. Being reminded and made aware of one´s own mortality constantly produced anxiety. Emotional pictures of coffins, mass graves, and patients on ventilators contributed to the collective fear. An excess of COVID-19 news generated anxiety and panic [87], also called “headline stress disorder” [88]. Negative social media news generates psychological stress that was unknown in former times and is well suited to cause mass hysteria [89]. Social media consumption correlates with anxiety and psychological distress [90]. Excessive discussion on the COVID-19 on social media deteriorated psychic health.

The news coverage of COVID-19 was almost completely negative. News on increasing COVID-19 cases outnumbered stories of declining cases by a factor of 5.5 even in times of falling cases [91]. News agencies may actually intentionally scare people [92] and suppress alternative information. In short, mass hysteria sponsored by a biased media sector may run out of control in a modern welfare state.

Fourth, negative news from an authoritative source produces anxiety and is particularly harmful for psychological health. Experts that endorse the credibility of a threat increase the spread of mass hysteria [93]. Many people, especially in times of crisis, look for help from the modern state. They attach great authority to the representatives of the state and to the warnings of state institutions. When doctors such as Anthony Fauci speak in the name of the state telling people that they face a terrible threat and have to wear masks and stay at home, it becomes easier for a collective hysteria to develop than would be the case in a more decentralized society where no such powerful central authority exists. Indeed, Doctor Fauci exaggerated the danger of COVID-19, delivering a message of panic to the public [94]. In a US congressional hearing on 11 March 2020, the mortality rate of the coronavirus was exaggerated. Information bias and selection bias led to the estimation that the mortality rate of the coronavirus would be ten times higher than the mortality rate of the seasonal influenza. There was a confusion of the case fatality rate, which is the proportion of deaths among confirmed cases of a disease and the infection fatality rate, which is the proportion of deaths relative to the prevalence of infections within a population. Estimations of infection fatality rates are based on blood tests. Estimated infections include undiagnosed, asymptomatic, and mild infections. The infection fatality rate is normally much lower than the case fatality rate. In the congressional hearing on 11 March 2020, the infection fatality rate of the seasonal influenza was compared to the estimated case fatality rate of the coronavirus, leading to the alarming statement that the coronavirus would be ten times more deadly than the seasonal influenza [95]. This false statement coming out of the Congress of the United States and with its authority greatly contributed to generate anxiety and panic.

Another factor that may make modern societies more receptible to mass hysterias is that the role of religion in society has been reduced. The fear of death is usually alleviated by religion because religions typically consider that there is a life after death. The state and democracy has been elevated to a quasi-religious level. The state appears as an alternative to God [96] without the promise of an afterlife. When turning away from religion, people start to fear death more, and a strong fear of death is another factor that contributes to panics, disorders, and mass hysteria [97]. As Erik von Kuehnelt-Leddihn has put it: “It is difficult to fear death if one is very pious. It is difficult not to worship health if one fears death. It is difficult to enforce general health without large scale state intervention and it is equally difficult to imagine increased state intervention without a loss of liberties.” [38,39,98] This does not mean that strong states never rely on religion. The authoritarian regimes of the interwar period, such as Austria with Dolfuß and Schuschnigg, Poland with Pilsudski, or Franco’s Spain, relied on religion and had characteristics of welfare states. However, empirical research found that in countries with higher welfare spending, people are less religious, suggesting that individuals substitute religious services for state services [99]. Religious beliefs, however, have been shown to increase psychological wellbeing [100]. Without a spiritual framework provided by religion and belief in an afterlife, there is a tendency for fear of death to increase and for the population to become more responsive to psychological problems and mass hysteria.

Fifth, the state may actively want to instill fear in the population, thereby contributing to the making of mass hysteria. Illustrating this point is the leakage of an internal paper of the German Department of the Interior during the first weeks of the COVID-19 crisis [101]. In the paper, the state experts recommended that the government should instill fear in the German population. In order to spread fear, the paper endorsed three communication strategies. First, the state authorities should stress the breathing problems of COVID-19 patients because human beings have a primordial fear of death by suffocation [102,103], which can easily trigger panic [104]. Second, the experts emphasized that fear should also be instilled in children, even though there is next to no risk to children´s own health. However, children could get easily infected by meeting and playing with other children. According to the report, children should be told that when they infect their parents and grandparents in turn, they could suffer a distressful death at home. This communication advice intended to invoke anxiety and feelings of guilt. Instilling guilt is another measure used by governments to make the population more supportive [105]. The recommended message instills fear of being responsible for infecting others who die a distressful death. Third, the German government was advised to mention the possibility of unknown long-term irreversible health damage caused by a SARS-CoV-2 infection and the possibility of a sudden and unexpected death of people who were infected. All these communication recommendations were intended to increase fear in the population. Fear, at the end, is an important foundation of a government’s power. As Henry H. Mencken put it: “the whole aim of practical politics is to keep the populace alarmed (and hence clamorous to be led to safety) by an endless series of hobgoblins, most of them imaginary.” [106] The overreaction of government to a perceived threat then fosters anxiety.

It lies in the interests of a government to emphasize citizens’ vulnerability to external and internal threats, because the state´s legitimacy and power rest on the narrative that it protects its citizens against such dangers. While the threat strategy is generally beneficial to the government, fear is a double-edged sword. Fear can also turn against the state. Panic and mass hysteria can even lead to total destabilization of a regime. Anecdotal evidence of this is the Grande Peur during the French Revolution, when rumors of aristocrats planning to starve the population led to general panic and uprisings against the regime.

Fear and anxiety have been an important factor in human evolution and have an important function. However, the evolutionary function of fear can be manipulated to secure dominion and control. Fear gives power over the fearful. The relationship between politics and fear has been widely studied [107,108,109]. The Ancient Greek historian Polybius claimed that in order to control the masses, rulers had to instill fear and work with horror images. The Roman writer Sallust pointed out that those who want to exercise power have to decide between generating fear or suffering from fear [110]. In recent times, the war against terror has been evoked by some authors as an example of reinforcing excessive fears in the population in order to increase the government’s power [111]. Brzezinski points out: “Constant reference to a ‘war on terror’ did accomplish one major objective: It stimulated the emergence of a culture of fear. Fear obscures reason, intensifies emotions and makes it easier for demagogic politicians to mobilize the public on behalf of the policies they want to pursue.” [112] A culture of fear [113,114] results from the government instilling fear in the general public to achieve its political goals, exploiting the negativity bias of the human brain.

Typically, governments are helped in their threat narrative by the media. As Robert Higgs points out: “the news media buy insurance against government retribution by playing along with whatever program of fear mongering the government is conducting currently.” [115] Sensationalist media also support the government’s fear strategy because they allow it to get the public’s attention. The combination of a state willing to use the fear strategy with supportive mass media provides fertile ground for mass hysteria to develop, with negative effects on public health.

Sixth, politicians have an incentive to overshoot the mark in their responses to a threat. This is because politicians are largely exempt from the risk of possible wrong decisions and their costs [116]. Political decision-makers can largely pass on the costs of their actions to others. Additionally, the larger and more centralized a state is, the better and more extensively the costs can be passed on to others [117,118].

Self-interested politicians [119,120] face an asymmetric pay-off. Underestimating a threat and failing to act has great political cost, as politicians will be held responsible for the disaster caused by the threat they underestimated. By contrast, an exaggeration or even invention of a threat and bold state intervention are politically more attractive. If the existential threat claimed by politicians really turns out to be such a great danger, they can be celebrated as heroes if they enacted bold measures. If the costs of these measures ultimately turn out to be excessive compared to the actual danger, then the politicians do not have to bear the cost of the wrong decision but can pass it on to the rest of the population. Politicians enjoying a guaranteed income therefore have an incentive to exaggerate a danger and to impose exaggerated measures, also called policy overreaction [121,122], which is conducive to the emergence and growth of mass hysteria.

In sum, property rights tend not to be effective limits in curbing mass hysteria in a welfare state. Moreover, the state may inhibit the natural mechanisms that reduce stress and hysteria. The centralized nature of the state increases group and conformity pressures. Politicized mass media and negative messages from official state agencies can further increase psychological pressure. Finally, the state may intentionally want to increase anxiety, and politicians have the incentive to make bold decisions and exaggerate the threat. Our findings are summarized in Table 3.

## 5. Conclusions

Mass hysteria can have enormous public health costs in terms of psychological stress, anxiety, and even physical symptoms. To these costs must be added indirect adverse health effects from alcoholism, suicides, or damage from deferred treatment and delayed recognition of illness. Policy failures in mass hysteria can lead to economic decline and poverty, which in turn negatively impacts public health and life expectancy.

Studies of mass hysteria have mostly focused on outbreaks in localized settings of schools or businesses. However, in the digital age of global mass and social media, the possibility of global mass hysteria exists, a phenomenon that has not yet been studied. Our study of the political economy of mass hysteria draws on the well-established psycho-logical phenomenon of mass hysteria and applies it to a new and innovative context of global mass hysteria for which no literature exists yet. More specifically, we analyzed how the political system can influence the likelihood and spread of mass hysteria in a digitized and globalized world based on economic principles. We discussed how the state and its size increase the likelihood of mass hysteria by comparing an idealized minimal state with an idealized welfare state, addressing a previously completely unexplored research question. Our findings are highly relevant and important because the policy failures induced by mass hysteria are potentially catastrophic for public health.

We found that the size and power of the state contributes positively to the likelihood and extensions of mass hysteria. The more centralized and the more power a state has, the higher the probability and extension of mass hysteria. In a minimal state, there exist self-correcting mechanisms that limit collective hysteria. The enforcement of private property rights limits the harm inflicted by those that succumb to the hysteria. The state (thanks to a fuzzy public sector and its soft power [123,124]), by contrast, amplifies and exacerbates mass panics, potentially causing important havoc. What are temporarily, locally limited, isolated outbreaks of mass hysteria, the state may convert into a global mass hysteria for an extended period of time. Recent development in information technology and, particularly, the use of social media, as well as a decline of religion, have made societies more prone to the development of mass hysteria [125,126,127]. Unfortunately, once a mass hysteria takes hold of the government, the amount of damage the hysteria can inflict to life and liberty surges as the state’s respect for private property and basic human rights is limited. The violation of basic human rights in the form of curfews, lockdowns, and coercive closure of business has been amply illustrated during the COVID-19 crisis. Naturally, the COVID-19 example is indicative rather than representative and its lessons cannot be generalized. During the COVID-19 crisis, several authors have argued that from a public health point of view, these invasive interventions such as lockdowns have been unnecessary [128,129,130,131] and, indeed, detrimental to overall public health [132,133]. In fact, prior scientific research on disease mitigation measures during a possible influenza pandemic had warned against such invasive interventions and recommended a more normal social functioning [134]. Moreover, in reaction to past pandemics such as the Asian flu of 1957–1958, there were no lockdowns [135], and research before 2020 had opposed lockdowns [136]. From this perspective, the lockdowns have been a policy error. We have shown that these policy errors may well have been produced by a collective hysteria. To which extent there has been a mass hysteria during the COVID-19 crisis is open for future research. In order to prevent the repetition of policy errors similar to those during the COVID-19 crisis, one should be aware of the political economy of mass hysteria developed in this article and the role of the state in fostering mass hysteria. Public health is likely to be affected negatively by state interventions during a mass hysteria due to policy errors.

## Figures and Tables

**Table 1:** COVID-19 survival rates per age in the US.

Age	Survival Rate
0–19 years	99.997%
20–49 years	99.98%
50–69 years	99.5%
70 + years	94.6%

Source: Centers for Disease Control and Prevention [52]. Own Calculations.

**Table 2:** Leading causes of death globally.

Disease	Deaths 2019 in Mio.
Ischemic heart disease	8.9
Stroke	6.1
Chronic obstructive pulmonary disease	3.3
Lower respiratory infections	2.6
Neonatal conditions	2.0
Trachea, bronchus, lung cancers	1.8
Alzheimer’s disease, other dementias	1.6
Diarrheal diseases	1.5
Diabetes mellitus	1.4
Kidney diseases	1.3

Source: WHO’s Global Health Estimates [54].

**Table 3:** The state’s impact on the development of mass hysteria.

Factors Influencing the Evolution of Mass Hysteria	Minimal State	Modern Welfare State
Stress and anxiety reducing strategies	function freely	can be severely restricted
Limits for produced harm	private property rights	insecure property rights
Possibility of experimentation with alternative solutions	facilitates discovery of real threat	centralization and group think inhibit alternative approaches
Politicized mass media	does not exist	likely to contribute to hysteria
Negative information from authoritative source	can contribute, but state is not regarded as responsible for public health	regarded as responsible for public health, high authority
Fear as a political factor	could be employed, but state power strictly limited	can be used to expand state power
Costs of wrong health decisions	Limited possibility to pass costs onto third parties	Extensive possibility to pass costs onto third parties, incentive to exaggerate threat

## Data Availability

Not applicable.
